# Kambin's triangle-related data based on magnetic resonance neurography and its role in percutaneous transforaminal endoscopic lumbar interbody fusion

**DOI:** 10.1186/s13018-022-03428-3

**Published:** 2022-12-15

**Authors:** Tianqi Li, Gang Wu, Yongle Dong, Zhiwei Song, Haijun Li

**Affiliations:** 1 Postgraduate Training Base of Dalian Medical University, Taizhou People’s Hospital, Jiangsu, 225300 China; 2 Postgraduate Training Base of Nantong University, Taizhou People’s Hospital, Jiangsu, 225300 China; 3 Department of Orthopedics, The Affiliated Taizhou People’s Hospital of Nanjing Medical University, Taizhou School of Clinical Medicine, Nanjing Medical University, 366 Taihu Road, Taizhou, 225300 Jiangsu China

**Keywords:** Kambin's triangle, Secure region, Positioning method, Magnetic resonance neurography of the lumbar nerve roots (MRN), Percutaneous transforaminal endoscopic lumbar interbody fusion (PETLIF)

## Abstract

**Background:**

The percutaneous transforaminal endoscopic lumbar interbody fusion (PETLIF) has many advantages as a new minimally invasive surgical technique, and its surgical approach passes through the Kambin's triangle. One of the greatest challenges in completing PETLIF is avoiding nerve root damage. In previous studies, the relevant anatomic data do not correspond well with current surgical techniques, and there is a paucity of studies based on magnetic resonance neurography (MRN), which is the clearest imaging method for nerve roots. The purpose of this study was to analyze the safety of the PETLIF at each lumbar segment based on measured results from the MRN imaging data and to propose a novel method of intraoperative positioning.

**Methods:**

The coronal images with the clearest course of nerve roots were chosen for retrospective observation. During the PETLIF, the secure region of the operation was considered to be a trapezium. The following parameters were measured, respectively: trapezoid area, height, and median line length, as well as the relevant parameters of the positional relation between the point “O,” the most secure operating center point of the secure region, and each osseous anatomic landmark. And the data were compared with the size of the cage to obtain safety.

**Results:**

At L1-S1, with the downward motion of the target intervertebral space, the area increased from (67.94 ± 15.22) mm^2^ to (140.99 ± 26.06) mm^2^, and the height increased from (7.23 ± 1.17) mm to (12.59 ± 1.63) mm. At L1–L5, the length of the median line was increased from (9.42 ± 1.70) mm to (12.70 ± 1.88) mm. Even though it was reduced to (11.59 ± 1.99) mm at L5–S1, it was still longer than that at L3–L4. The safety obtained by the primary observational indicator was 34.52%, 33.33%, 53.57%, 96.43%, and 77.38%, respectively, at L1–S1. The safety obtained by the two secondary observational indicators was 77.38% and 95.24% at L3–L4 and 100% at L4–S1. There was no point “O” outside the anatomic mark line. The intraoperative positioning method of the point “O” was as follows: It was located medially and horizontally approximately 3/5 of the anatomic mark line at L1–L5; the horizontal distances were (0.48 ± 0.67) mm, (1.20 ± 0.89) mm, (2.72 ± 1.01) mm, and (3.69 ± 1.47) mm, respectively. In addition, it was necessary to locate (3.43 ± 1.41) mm inward at about 4/5 of the anatomic mark line at L5–S1.

**Conclusions:**

The MRN allows clearer and more accurate visualization of the nerve roots, and the basic anatomic study of the Kambin's triangle based on this technology is of practical clinical significance. In the current study, it is believed that, during the PETLIF, cage implantation is the safest at L4–L5, followed by L5–S1; L1–L3 is more likely to cause nerve root injury, and L3–L4 is not less likely. To improve safety, a comprehensive individualized imaging assessment should be performed before surgery. This study also provides an easy method of intraoperative localization, which helps avoid nerve root injury.

## Background

Lumbar interbody fusion, one of the surgical approaches for the treatment of lumbar instability, has been widely used in the treatment of various spinal diseases [1]. Surgeons continue to improve the surgical approach, but the incidence of complications related to surgery remains high due to the invasive nature of open surgery [2]. In addition to bringing great pain to patients, the occurrence of postoperative complications increases the additional medical burden on society. So, scholars at home and abroad continue to pursue more minimally invasive surgical methods for lumbar interbody fusion in an effort to reduce the incidence of surgical complications.

The extensive study of spinal anatomy by surgeons, the continued refinement of surgical methods, and the invention of surgical instruments have led to the rise of minimally invasive spinal surgery techniques. The surgical technique of the minimally invasive surgery transforaminal lumbar interbody fusion (MIS-TLIF) proposed by Foley et al. has significant minimally invasive advantages over previous open surgical techniques that can reduce intraoperative blood loss, the patient recovery time, and the length of hospital stay [3–5]. At the same time, endoscopic technology has been introduced into the field of spine surgery, and the widespread application of the surgical technique, the percutaneous transforaminal endoscopic lumbar discectomy (PELD), for nerve root decompression with endoscopic assistance to resection of the herniated nucleus pulposus with open-vision has resulted in spinal surgeons becoming more proficient in the establishment of endoscopic operating channels and the use of endoscopes [6]. The anterior lumbar fusion cage was also large and difficult to place into the intervertebral space via a narrow endoscopic operating channel, and the invention of the expandable fusion cage has overcome this difficulty [7, 8]. The percutaneous transforaminal endoscopic lumbar interbody fusion (PETLIF) is an alternative method of lumbar interbody fusion due to the combination of MIS-TLIF and PELD surgical techniques as well as the application of the expandable fusion cage.

The surgical approach of the PETLIF is via the Kambin's triangle, an anatomic corridor that was proposed by Kambin et al. in 1987, which is a triangular area located below the nerve root on the posterolateral side of the lumbar spine, and there are no important anatomic structures such as blood vessels and nerves in the corridor, so it has become a safe working region for surgical operations. At present, it is recognized that the Kambin's triangle is a three-dimensional spatial structure consisting of four boundaries, of which the medial boundary consists of the dural sac and a part of the traversing nerve root, the lateral boundary is the exiting nerve root, the lower boundary is the upper endplate plane of the vertebral body below the target intervertebral space, and the posterior boundary is the superior articular process (SAP) of the vertebral body beneath the target intervertebral space [9, 10]. The three-dimensional anatomical channel of the Kambin’s triangle projects onto the coronal plane as a triangular region consisting of the outer edge of the dural sac and a part of the traversing nerve root, the inner edge of the exiting nerve root, and the horizontal line of the superior endplate of the inferior vertebral body. On the sagittal plane, Min et al. believed the actual working region to be a trapezoidal-like area consisting of the posterior edge of the exiting nerve root, the anterior edge of the SAP, and the horizontal line of the superior and inferior vertebral endplates of the target intervertebral space [11].

Since the Kambin's triangle is an anatomically inherent channel of the human body, the surgical technology of the PETLIF leverages this anatomic advantage to perform minimally invasive surgical procedures under endoscope-assisted direct vision, which theoretically can combine the benefits of minimally invasive fusion surgery and endoscopic spinal surgery, while at the same time preserving the stable structure of the posterior portion of the spine, to avoid iatrogenic injury caused by surgery as much as possible, to reduce the incidence of surgical complications, to contribute to the restoration of biomechanical stability, and to expedite patient recovery. Recently, the PETLIF has been shown to have a minimal invasive advantage over MIS-TLIF, which can significantly reduce intraoperative blood loss and hospital length of stay while still achieving good early clinical outcomes, and also has some benefits in alleviating postoperative low back pain and hastening patient recovery [12–14]. In addition, the study by Wang MY et al. reported that the PETLIF can be performed without generalized anesthesia, by injecting long-lasting (liposomal) bupivacaine along the surgical trajectory for local analgesia prior to incision, and only by a continuous infusion of propofol and ketamine to keep the patient under light to moderate sedation. These awake spine surgeries allow surgeons to benefit from real-time neuromonitoring and patient feedback, which may indicate the proximity of the instruments to critical neural elements, and that the absence of general anesthesia can eliminate its associated risks, particularly for older or significantly ill patients. [15, 16]. The PETLIF has the potential to meet the needs of minimally invasive spine surgery development and the requirements of the ERAS concept (enhanced recovery after surgery) and is an ideal surgical approach.

The greatest challenge in completing the PETLIF is the limited surgical operating space between the traversing nerve root and the exiting nerve root, and the precise location of the boundary of the Kambin's triangle and the specific location of the nerve root cannot be precisely located during the procedure. If the intraoperative operation deviates too far from the Kambin's triangle, exposure of the Kambin's triangle with the circular saw or cage implantation can easily cause damage to the nerve roots. Clarifying the position of nerve roots and the relative relationship with adjacent anatomical structures has practical clinical significance to protect nerve roots during operation [17]. For practical clinical applications, there is a lack of a method that can precisely locate the center of the Kambin's triangle with the aid of intraoperative fluoroscopy via known anatomic landmarks. The pedicle is a commonly used anatomic positioning landmark for fluoroscopy in spinal surgery, which is easily obtained. If the relative position of the center of the Kambin's triangle concerning the projection of the pedicles can be obtained preoperatively, this can be used to guide the surgical operation. A preoperative imaging examination such as magnetic resonance neurography (MRN) may help us to obtain the anatomic relationship described above.

On the other hand, if the surgical instrument or the interbody fusion cage is oversized, the nerve root injury can occur even if it is precisely entered into the intervertebral space from the center of the Kambin's triangle during the surgical procedure [18]. Accurate anatomic data from the Kambin's triangle at each segment of the lumbar spine are thus an important basis for judging whether or not the interbody fusion cage can be safely implanted. The specific operation of lumbar interbody fusion through the intervertebral foramen using endoscopy was not the same as that performed in the previous studies, and there were various understandings of the definition and boundaries of the Kambin’s triangle [9, 19, 20]. It should be noted that the anatomic parameters measured in previous studies do not correspond exactly to the current surgical techniques and cannot be used directly to guide the PETLIF. Thus, for PETLIF to develop into a full-fledged surgical method, accurate basic anatomic data related to the Kambin's triangle that matches this technique must be obtained to guide surgical procedures.

This study is based on the imaging technology of the MRN of the lumbar nerve roots. This imaging technology can more clearly show the course of nerve roots and the relationship between nerve roots and bony anatomical landmarks [21, 22]. The measurement in this study allows us to obtain accurate related anatomic data of the Kambin's triangle corresponding to the current surgical technique of PETLIF, and to compare it with the size of the expandable fusion cage applied during surgery to judge whether each lumbar segment can be safely implanted with the cage. It also provides an anatomic reference for the development of novel surgical instruments suitable for PETLIF. In addition, we discuss the relationship between the safest point of positioning for intervertebral space access and the osseous anatomical landmarks during the PETLIF, providing a novel method of intraoperative localization for the PETLIF to decrease the risk of intraoperative nerve root injury.

## Patients and methods

This study was approved by the ethics committee of Taizhou People's Hospital (ethics approval No. KY202201501). Using the HIS system (Neusoft, China), the data from patients with low back pain who required MRN of the lumbar nerve roots for differential diagnosis at Taizhou People's Hospital from April 1, 2017, to December 31, 2021, were collected retrospectively. The basic information in the patient's medical records (including name, gender, age, height, weight, and anamnesis) was collected, and the imaging data were screened according to the inclusion and exclusion criteria.

Imaging data were acquired by MAGNETOM Skyra 3.0 T (SIEMENS, Germany) machine scanning, 3D double echo steady-state MRI sequence (T2W1 3D-DESS) was used as the basic sequence on the coronal plane, combined with T2W1 sagittal image to scan and localize the nerve roots in the target area, and the trailing edge of the positioning line covered 3–5 layers of the posterior edge of the spinal canal. The coronal images were taken from the upper edge of the T12-L1 intervertebral disk to the lower edge of the S1 vertebral body. The images were post-processed using syngo. Via (SIEMENS, Germany). The PACS/RIS system (Neusoft, China) was used for retrospective observation, the level with the clearest exiting nerve root course in the L1-L2 to L5-S1 intervertebral space segment was selected, and the basic anatomic data related to the Kambin's triangle were measured on the coronal images.

### Inclusion and exclusion criteria

*Inclusion criteria*: Adults between the ages of 18 and 70 years;

*Exclusion criteria*: Lumbar fracture; traumatic lumbar spondylolisthesis; congenital spinal deformity; scoliosis and kyphosis; lumbosacral transitional vertebrae; metabolic bone disease; severe spinal instability; severe stenosis of the intervertebral foramen; severe degenerative lesions; history of previous lumbar surgery, spinal tumor, spinal tuberculosis, and spinal infection; imaging data demonstrated significant changes in the course of the nerve roots.

### Measuring method

#### Definition of the secure region of the operation during PETLIF procedures and the method of measuring the relevant anatomic data

**Fig. 1:**
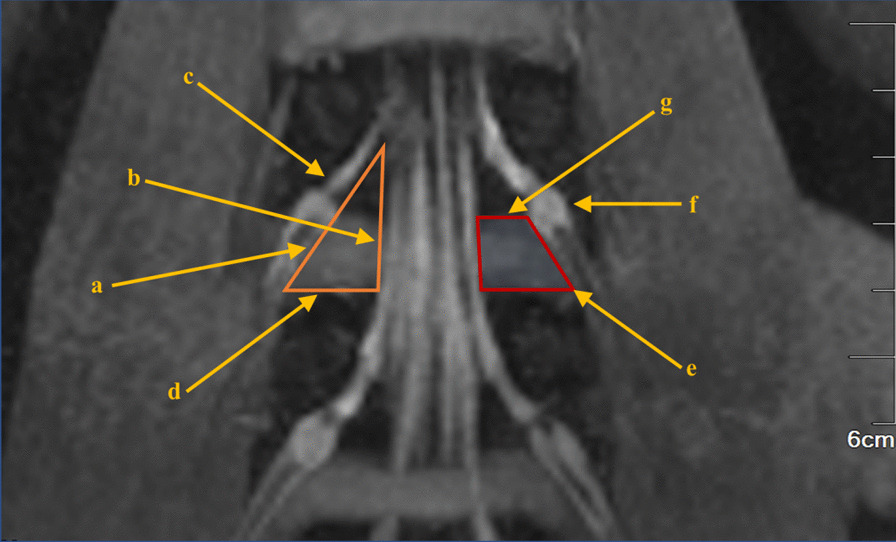
Schematic diagram of the Kambin's triangle and the secure region on the coronal plane of the MRN of the lumbar nerve roots. a. Coronal projection of the Kambin's triangle; b. the dural sac and a part of the traversing nerve root; c. the exiting nerve root; d. the upper endplate of the inferior vertebral body of the target intervertebral space; e. the secure region of the operation during PETLIF procedures; f. the ganglion of exiting nerve root; g. the lower endplate of the superior vertebral body of the target intervertebral space.

The projection of the Kambin's triangle on the coronal plane is intersected with the horizontal line of the inferior endplate of the superior vertebral body at the level of the target intervertebral space, and the trapezoid below the horizontal line is defined to be the secure region of the operation during the PETLIF procedures. The top and bottom boundaries of the secure region correspond to the horizontal line of the lower endplate of the superior vertebral body and the upper endplate of the inferior vertebral body of the target intervertebral space, respectively. The medial margin of the exiting nerve root is taken as the lateral boundary. The lateral margin of the dural sac and part of the traversing nerve root is measured as the medial boundary (Fig. 1).

**Fig. 2:**
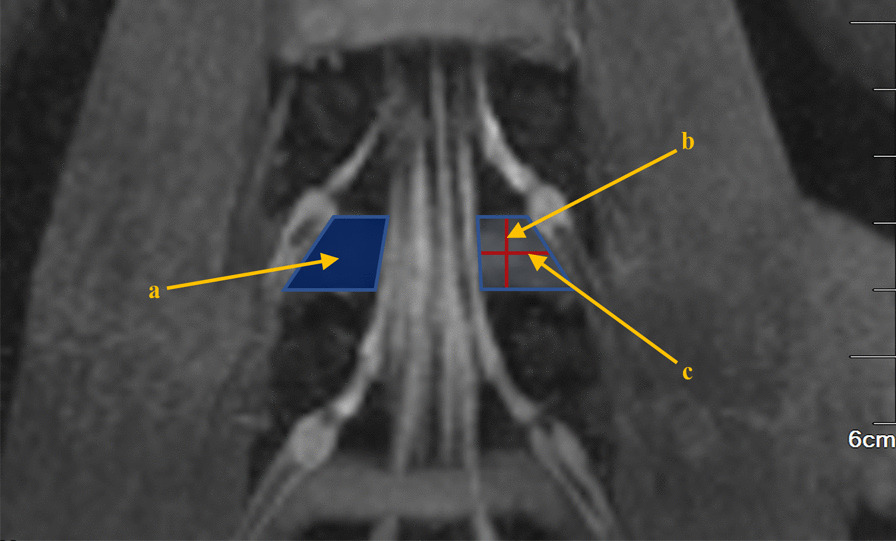
Schematic diagram of the area, height, and width of the secure region. (a. The area of the secure region; b. the height of the secure region; c. the width of the secure region.)

The length of the line connecting the midpoints of the two waists of the trapezoid, the length of the median line of the trapezoid, is defined as the width of the secure region (Fig. 2). For each patient, measurements were made of the area, height, and width of the secure region on the left and right sides of each intervertebral space from L1-L2 to L5-S1.

#### Observational indicators of safe placement of the expandable fusion cage during the PETLIF procedures

In the case of the PETLIF at Taizhou People's Hospital, the cage used is the height-adjustable expandable fusion cage (Shanghai Reach Medical Instrument Co., Ltd., Shanghai, China). The secure region data obtained were compared to the dimensions of this cage before expansion: The width of the secure region as the primary observational indicator compared to the width (10 mm) of the cage. As secondary observational indicators, the area and the height of the secure region were compared to the minimum cross-sectional area (80 mm^2^) and the height before expansion (8 mm) of the cage, respectively, to assess the safety of cage implantation during the PETLIF for various segments of the lumbar intervertebral space.

The samples with a width of the secure region longer than the width of the cage are classified as the safety group (Group A), which have a low risk of nerve root injury during cage implantation and there is no need to be overly concerned about nerve root injury during the PETLIF. The samples with a width of the secure region equal to or less than the width of the cage are classified as the unsafe group (Group B), which will have an increased risk of nerve root injury when implanted with the cage. In the Group B, due to the narrow space, a certain degree of traction of the nerve roots is necessary during the procedure, and efforts should be made to avoid nerve root injury when performing PETLIF surgery.

Given that the sample size of Group A corresponds to the sample size of the intervertebral foramina where the cage can be safely implanted, the sample size of group A from each intervertebral space (n) was divided by the total sample size from the same intervertebral space (N) to obtain a ratio directly proportional to the safety of cage implantation at the target intervertebral space segment, which is expressed as a percentage in this study, as shown in formula (1). Comparing each intervertebral space, the higher the ratio, the less likely it is that the nerve root will be damaged during cage implantation, that is, the higher the safety. In this study, we evaluated the risk of nerve roots injury when the cage is implanted in each lumbar intervertebral space, and safety ≤ 40% was regarded as high risk, safety > 40% and ≤ 70% as medium risk and safety > 70% as low risk. Likewise, for the two secondary observational indicators of the area and height, this ratio also represents the safety of cage implantation at the target intervertebral space as a percentage.

1 $${\text{Formula:\, the safety }}\left( {\text{\% }} \right) = \frac{n}{N}\%$$

#### The method of measurement and the definition of measurement-related parameters of the relative positional relationship between various osseous anatomical landmarks and the most secure operating center point during PETLIF procedures

The midpoint of the median line of the trapezoid is defined as the center point “O” of the secure region, and there is a certain space of operations around this point. Theoretically, taking the point “O” as the target point for operation channel insertion can reduce the risk of nerve root injury during both the process of using a circular saw to expose the Kambin's triangle under the blind vision and the implantation of the expandable fusion cage.

**Fig. 3:**
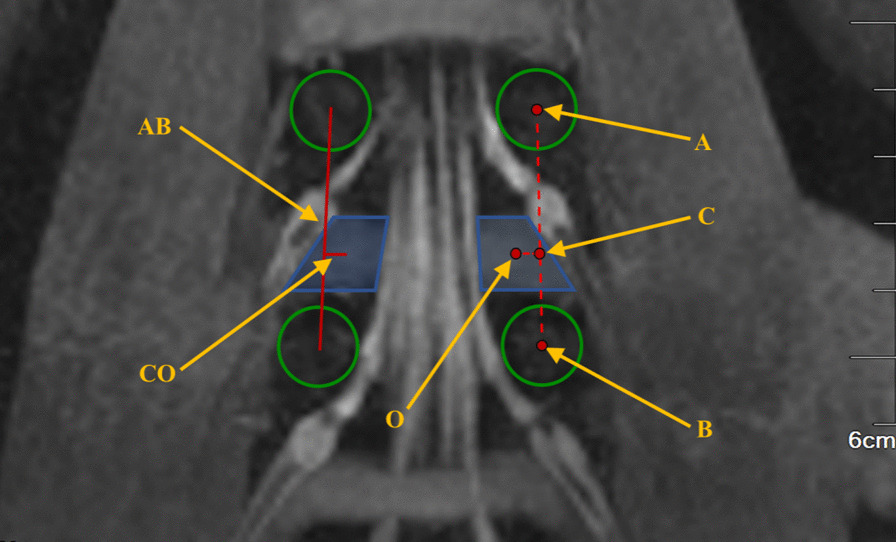
Relationship of position between the most secure operating center point and the osseous anatomical landmarks at L1-L5. (A. Central point of the ipsilateral pedicle of the superior vertebral body at intervertebral space; B. central point of the ipsilateral pedicle of the inferior vertebral body at intervertebral space; C. the intersection of the horizontal line of the point “O” and the line segment “AB”; O. the most secure operating center point during the PETLIF procedures.)

For the four intervertebral spaces from L1–L2 to L4–L5, the center of the pedicle on the measuring side of the superior vertebral body at the target intervertebral space, the pedicle “eye,” is defined as point “A,” and the pedicle center of the ipsilateral inferior vertebral body is defined as point “B.” The two points are connected by a line segment “AB,” which is defined as the anatomic mark line at the L1–L5. The intersection of the horizontal line of the point “O” and the line segment “AB” is defined as point “C,” and the length of the line segment “CO” is the horizontal distance from the point “O” to the anatomic mark line. Measure the length of line segment “CO,” and then calculate the ratio of the length of line segment “AC” to the length of line segment “AB.” These data are used to describe the relative positional relationship between the point “O” and the pedicles at each target intervertebral space (Fig. 3).

**Fig. 4:**
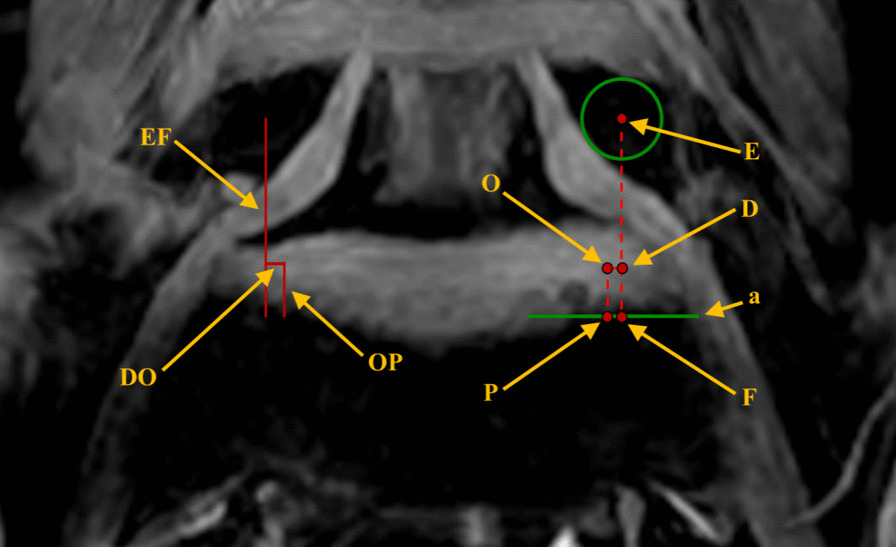
Relationship of position between the most secure operating center point and the osseous anatomical landmarks at L5-S1. (E. The central point of the ipsilateral L5 pedicle; F. The intersection of the upper edge of the sacrum and the vertical line passing through the central point of the L5 pedicle; D. The intersection of the horizontal line of the point “O” and the line segment “EF”; P. The intersection of the upper edge of the sacrum and the vertical line passing through the point “O”; O. The most secure operating center point during the PETLIF procedures; a. The horizontal line of the superior margin of the sacrum.)

In the case of the L5–S1 intervertebral space, the center of the pedicle on the measuring side of the L5 vertebral body is defined as point “E.” Draw a vertical line through the point “E,” and the intersection of the vertical line with the upper edge of the sacrum is defined to be point “F.” The two points are connected as a line segment “EF,” which is defined as the anatomic mark line at L5–S1. The intersection of the line segment “EF” and the horizontal line of the point “O” is defined to be the point “D,” and the length of the line segment “DO” is the horizontal distance from point “O” to the anatomical mark line. The vertical line of point “O” intersects the upper edge of the sacrum at point “P.” Measure the length of the line segment “DO,” and then calculate the ratio of the length of line segment “ED” to the length of line segment “EF.” Finally, measure the distance from point “O” to the upper edge of the sacrum, that is, the length of line segment “OP.” These data are used to describe the relative positional relationship between the point “O” to the L5 pedicle ipsilateral and the upper edge of the sacrum at L5–S1 (Fig. 4).

### Statistical analysis

Data from 42 patients were measured, and the quantitative normal distribution data described the characteristics of the measurement by mean ± standard deviation (Mean ± SD). Classification data were represented as counts (percentage): n (%). Statistical analysis was performed using SPSS 26.0 (IBM, USA). Once the measurement data passed the homogeneity of variance test, the mean and standard deviation were obtained by one-way analysis of variance (ANOVA), and the 95% confidence interval was calculated. Multiple comparisons with the least significant difference (LSD) were performed following the analysis of variance, Bonferroni and Dunnett’s tests were used to test whether there was a significant difference between the sample mean of each segment of intervertebral space. A paired t test or rank sum test was used to compare measured values on the left and right depending on whether the data were normally distributed. Chi-square test was used for independent samples of the classified data. *P* < 0.05 is considered statistically significant.

## Results

### Demographical characteristics and clinical data of the patients

Patients' baseline medical records and imaging data were retrospectively reviewed. According to the inclusion and exclusion criteria, a total of 42 patients (21 males and 21 females) were enrolled in the present study. The nerve roots in the imaging data of 42 patients were well-imaged. The demographic characteristics and clinical information of the included patients are given in Table 1.

**Table 1:** Demographical and clinical characteristics of the patients

Characteristic	Patients (n = 42)
Age (yrs)	50.07 ± 11.23
*Sex (no. of patients/%)*	
Male	21 (50)
Female	21 (50)
BMI(kg/m^2^)	23.75 ± 3.10
*Complication (no. of patients/%)*	
Hypertension	5 (11.90)
Diabetes	2 (4.76)
Cardiovascular disease	2 (4.76)

Age and BMI are expressed as mean ± SD

The remaining characteristics are expressed in terms of the number of patients (the percentage of the total number of patients)

### The area, height, and width of the secure region

**Fig. 5:**
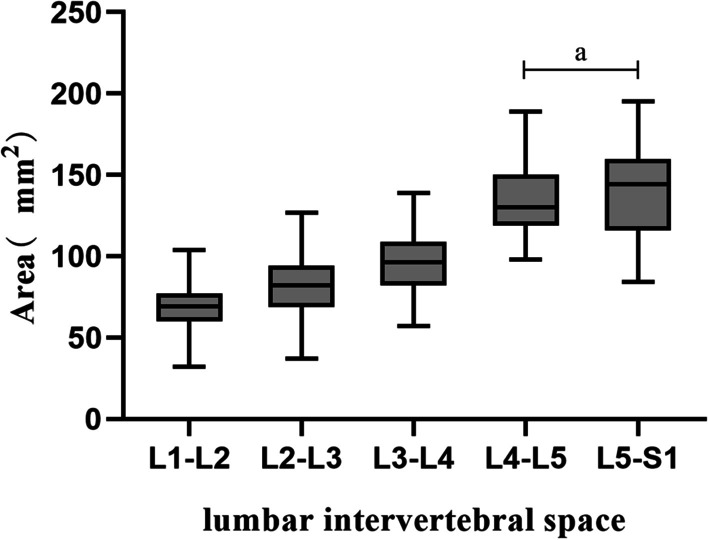
Area of the secure region at each lumbar intervertebral space. With “a” indicating no significant difference between the two groups (*P* > 0.05)

There was a gradual increase in the mean area value of the secure region from L1–L2 to L5-S1 which is the smallest at the L1–L2 intervertebral space, which was found to be (67.94 ± 15.22) mm^2^. The area at L4–L5 and L5–S1 was (135.44 ± 23.38) mm^2^ and (140.99 ± 26.06) mm^2^, respectively, and there was no significant difference between them (P > 0.05). A comparison of the remaining segments of the intervertebral space revealed a statistically significant difference (*P* < 0.001), as given in Table 2 (Fig. 5).

**Table 2:** Relevant parameters of the area, height, and width of the secure region

Level (side)	Area (mm^2^)		Height (mm)		Width (mm)	
	Mean ± SD	95% CI	Mean ± SD	95% CI	Mean ± SD	95% CI
*L1-L2*						
Right	68.29 ± 15.38	63.49–73.08	7.22 ± 1.19	6.85–7.59	9.50 ± 1.65	8.98–10.02
Left	67.60 ± 15.23	62.85–72.34	7.25 ± 1.16	6.89–7.61	9.34 ± 1.76	8.79–9.89
Mean	67.94 ± 15.22	64.64–71.24	7.23 ± 1.17	6.98–7.49	9.42 ± 1.70	9.05–9.79
*L2-L3*						
Right	81.19 ± 16.78	75.96–85.42	8.48 ± 0.96	8.18–8.78	9.74 ± 1.79	9.19–10.30
Left	81.40 ± 15.56	76.55–86.25	8.49 ± 0.98	8.18–8.80	9.63 ± 1.67	9.11–10.15
Mean	81.30 ± 16.09	77.81–84.79	8.49 ± 0.97	8.28–8.70	9.69 ± 1.72	9.32–10.06
*L3-L4*						
Right	96.88 ± 19.76	90.72–103.04	9.63 ± 1.00	9.32–9.94	10.24 ± 1.59	9.75–10.73
Left	96.02 ± 19.23	90.03–102.02	9.65 ± 0.96	9.35–9.95	10.20 ± 1.69	9.67–10.72
Mean	96.45 ± 19.38	92.25–100.66	9.64 ± 0.98	9.43–9.85	10.22 ± 1.63	9.86–10.57
*L4-L5*						
Right	137.12 ± 23.62	129.76–144.48	10.87 ± 1.37	10.44–11.30	12.83 ± 1.86	12.25–13.40
Left	133.76 ± 23.30	126.50–141.02	10.96 ± 1.35	10.54–11.38	12.57 ± 1.91	11.97–13.16
Mean	135.44 ± 23.38	130.37–140.51	10.92 ± 1.35	10.62–11.21	12.70 ± 1.88	12.29–13.10
*L5-S1*						
Right	142.57 ± 27.07	134.14–151.01	12.55 ± 1.67	12.03–13.07	11.86 ± 2.10	11.20–12.51
Left	139.40 ± 25.24	131.54–147.27	12.63 ± 1.62	12.13–13.14	11.33 ± 1.85	10.75–11.90
Mean	140.99 ± 26.06	135.33–146.64	12.59 ± 1.63	12.23–12.94	11.59 ± 1.99	11.16–12.02

Values are expressed as mean ± SD

With a total sample size of 84

**Fig. 6:**
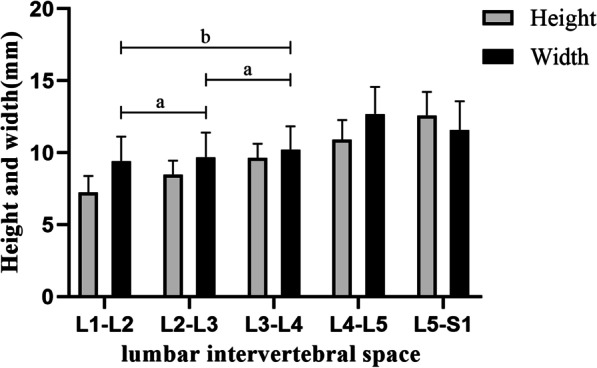
Bar chart of the height and width of the secure region at each lumbar intervertebral space. With “a” representing the comparison between the two groups (*P* > 0.05). With “b” representing the comparison between the two groups (*P* < 0.05 and > 0.001)

**Fig. 7:**
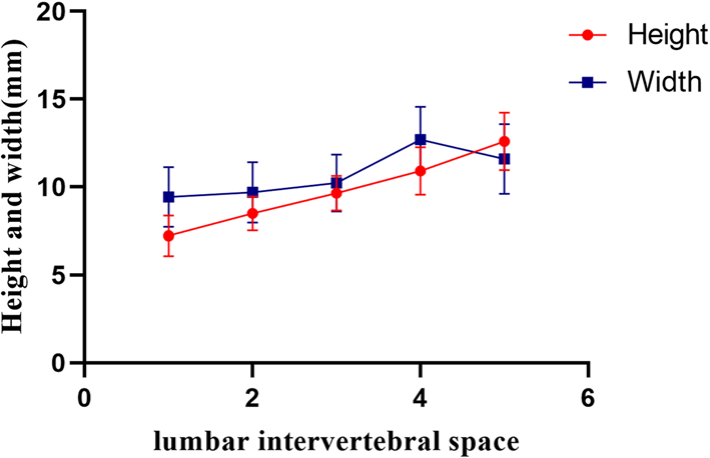
Broken line plot of the height and width of the secure region at each lumbar intervertebral space

From L1-2 to L5-S1 intervertebral space, there was a gradual increase in the average height value of the secure region, ranging in size from (7.23 ± 1.17) mm to (12.59 ± 1.63) mm, as given in Table 2. The statistical difference between the segments within each intervertebral space was highly statistically significant (*P* < 0.001) (Fig. 6 and Fig. 7).

The mean value of the width of the secure region gradually increased from L1–L2 to L4–L5, and L5–S1 decreased compared with the L4-L5. The width at L1–L2 was the shortest with an average value of (9.42 ± 1.70) mm, and the width at L4–L5 reached the maximum (12.70 ± 1.88) mm. Even though the width at L5–S1 was decreased to (11.59 ± 1.99) mm, this length is still longer than (10.22 ± 1.63) mm at L3–L4, as presented in Table 2. The difference between L2–L3 and its superior and inferior segments of intervertebral space was not statistically significant (*P* > 0.05). There was a statistical difference between L1–L2 and L3–L4 intervertebral space segments (*P* < 0.05, *P* > 0.001). The remaining intervertebral spaces were statistically significant differences from each other (*P* < 0.001) (Figs. 6 and 7).

In terms of the area, height, or width of the secure region, there was no statistically significant difference between the left side and the right side of each intervertebral space (*P* > 0.05).

### Results related to the safety of cage implantation during PETLIF procedures

In each intervertebral space at L1–S1, 84 groups of data related to the size of the secure region were measured. We compared the area, height, and width of the secure region to the corresponding size data of the cage. A comparison of the safety of implantation of an expandable fusion cage into each segment of intervertebral space revealed that the differences were statistically significant (*P* < 0.05).

**Fig. 8:**
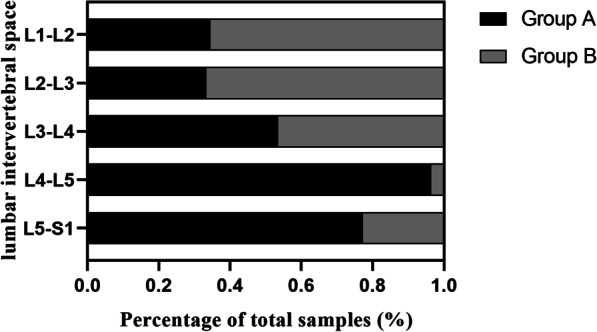
Percentage of the sample size of Group A and Group B is classified by the width of the secure region in the total sample size at each lumbar intervertebral space. The samples with the width of the secure region longer than the width of the cage were classified into the safety group (Group A); the other samples were classified as the unsafe group (Group B)

In this study, the width of the secure region was used as the primary observational indicator. Relative to the width of the secure region and the width of the cage. Fewer samples can be safely implanted with the cage at L1–L2 and L2–L3 is less, 29 and 28 samples, respectively, and the safety was 34.52% and 33.33%, respectively. The risk of nerve root damage when inserting a fusion cage is high at L1–L2 and L2–L3. In comparison with the other lumbar intervertebral spaces, the L4–L5 segment of the intervertebral space had the highest safety for cage implantation, which was 96.43%. Although the safety at L5–S1 (77.38%) was less than that at L4–L5, it remains safe to implant the cage. When the cage was implanted in the L3–L4 intervertebral space segment, the risk of nerve root injury was medium, and its safety was 53.57%, as presented in Table 3 (Fig. 8).

**Table 3:** Safety of the expandable fusion cage implantation

Level	Area (%/n)	Height (%/n)	Width (%/n)
L1-L2	13.10 (11)	23.81 (20)	34.52 (29)
L2-L3	55.95 (47)	79.76 (67)	33.33 (28)
L3-L4	77.38 (65)	95.24 (80)	53.57 (45)
L4-L5	100 (84)	100 (84)	96.43 (81)
L5-S1	100 (84)	100 (84)	77.38 (65)

Values are expressed as the ratio of the sample size that the measured parameters are larger than the corresponding cage size to the total sample size at each segment (the number of samples that the measured parameters are larger than the corresponding size of the cage)

The risk of nerve root injury during cage implantation in each lumbar intervertebral space was assessed based on the safety of the three observational indicators of area, height, and width, and safety ≤ 40% was considered high risk, safety > 40% and ≤ 70% medium risk, and safety > 70% low risk

**Fig. 9:**
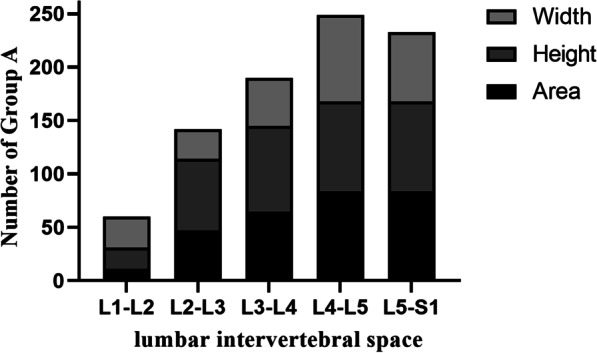
Sample size accumulation plot of Group A classified according to the three observational indicators at each lumbar intervertebral space

The area and height of the secure region were taken as the secondary observational indicators, which were compared to the minimum cross-sectional area and height of the cage, respectively. Both of these two observational indicators indicated that the risk of nerve root injury when inserting the cage at L1–L2 was high, and the safety was 13.10% and 23.81%, respectively, and the safety at L3–L4 was 77.38% and 95.24%, respectively, which was low risk. For all samples at L4–5 and L5–S1, the measured data regarding these two observational indicators were greater than the relevant data on the size of the expandable fusion cage, as presented in Table 3 (Fig. 9).

### The relative positional relationship between various osseous anatomical landmarks and the most secure operating center point during PETLIF procedures

The anatomic mark line of each intervertebral space at the L1–L5 was divided into upper and lower portions by the horizontal line of the point “O.” For the four intervertebral spaces from L1–L2 to L4–L5, the ratio of the length of the upper half part of the anatomic mark line to the total length gradually increased with the downward movement of the target intervertebral space segment, that is, the ratio of “AC” to “AB” gradually increased, as presented in Table 4.

**Table 4:** Positional relationship between the most secure operating center point and the center of upper and lower pedicles at each segment

Level (side)	AC/AB		CO (mm)	
	Mean ± SD	95% CI	Mean ± SD	95% CI
*L1-L2*				
Right	0.58 ± 0.04	0.57–0.59	0.37 ± 0.64	0.18–0.57
Left	0.57 ± 0.04	0.56–0.58	0.58 ± 0.69	0.37–0.80
Mean	0.57 ± 0.04	0.56–0.58	0.48 ± 0.67	0.33–0.62
*L2-L3*				
Right	0.60 ± 0.03	0.59–0.61	1.03 ± 0.79	0.79–1.28
Left	0.59 ± 0.03	0.58–0.60	1.37 ± 0.97	1.07–1.67
Mean	0.60 ± 0.03	0.59–0.60	1.20 ± 0.89	1.01–1.40
*L3-L4*				
Right	0.62 ± 0.04	0.60–0.63	2.52 ± 1.04	2.19–2.84
Left	0.61 ± 0.04	0.60–0.62	2.92 ± 0.95	2.62–3.22
Mean	0.62 ± 0.04	0.61–0.62	2.72 ± 1.01	2.50–2.94
*L4-L5*				
Right	0.63 ± 0.04	0.62–0.64	3.64 ± 1.45	3.18–4.09
Left	0.63 ± 0.04	0.62–0.64	3.74 ± 1.50	3.27–4.21
Mean	0.63 ± 0.04	0.62–0.64	3.69 ± 1.47	3.37–4.01

The point “O” represents the most secure operating center during the PETLIF procedure; the point “C” represents the intersection of the horizontal line of the point “O” with the line connecting the center points of the upper and lower pedicles; values of the “AC/AB” are the ratio of the distance from the point “C” to the center point of the upper pedicle to the distance between the center points of the upper and lower pedicles; values of the “CO” are the horizontal distance from the point “O” to the line connecting the center points of the upper and lower pedicles

**Fig. 10:**
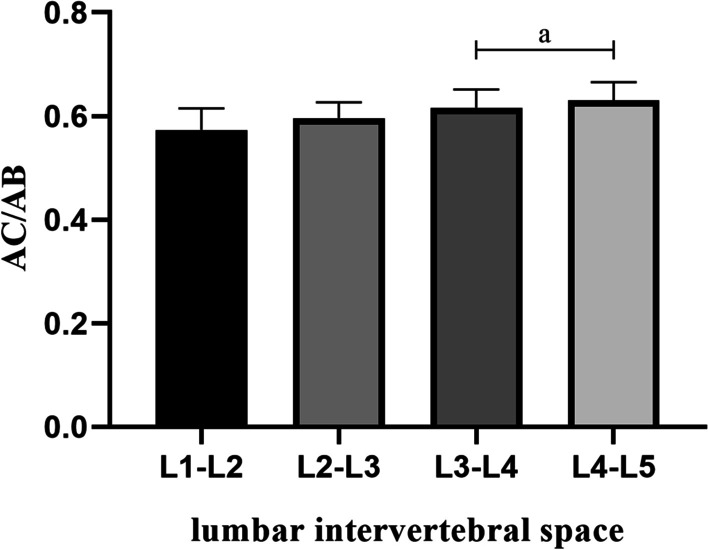
Ratio of the length of the upper half part of the anatomic mark line to the total length at each segment of the lumbar intervertebral space of L1–L5. With “a” representing the comparison between the two groups (*P* < 0.05, *P* > 0.001)

The difference between L3–4 and L4–L5 was found to be statistically significant (*P* < 0.05, *P* > 0.001), and there was a highly statistically significant difference between the remaining intervertebral spaces (*P* < 0.001). The ratio was the smallest at L1–L2, which was (0.57 ± 0.04); (0.60 ± 0.03) at L2–L3; (0.62 ± 0.04) at L3–L4; the ratio peaked at L4-L5 which was found to be (0.63 ± 0.04) (Fig. 10).

**Fig. 11:**
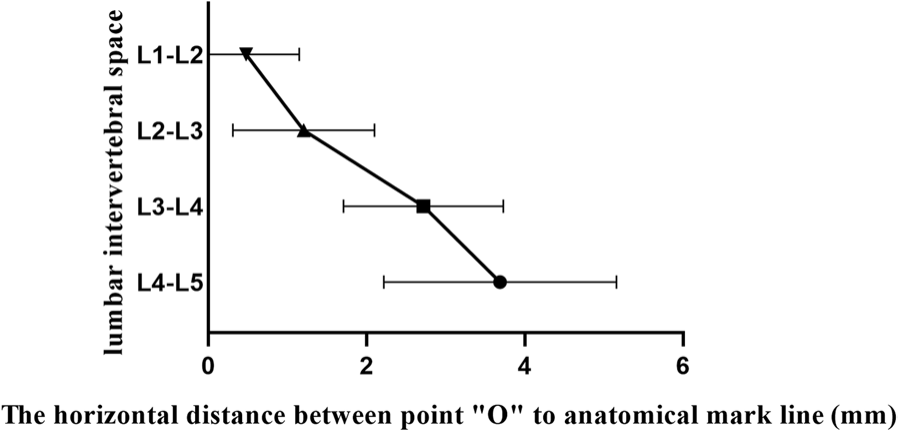
Mean value of the horizontal distance from the point “O” to the anatomic mark line at each segment of the lumbar intervertebral space of L1–L5

For the four intervertebral spaces from L1–L2 to L4–L5, it was not observed that the most secure operating center point was not observed to be outside the anatomical mark line, that is, all of the point “O” were within or coincides with the anatomic mark lines. The average horizontal distance from the point “O” to the anatomic mark line gradually increased with the downward movement of the target intervertebral space segment. In other words, the length of the line segment “CO” became longer and longer, and its mean value gradually increased from (0.48 ± 0.67) mm at L1–L2 to (3.69 ± 1.47) mm at L4–L5. Statistically significant differences were observed between the four intervertebral spaces from L1–L2 to L4–L5 (*P* < 0.001), as presented in Table 4 (Fig. 11).

For the four intervertebral spaces from L1–L2 to L4–L5, there was no significant difference in the parameters between the left and right sides of each intervertebral space (*P* > 0.05).

At the L5-S1, the mean value of the ratio of the length of the line segment “ED” to the length of the line segment “EF” was (0.77 ± 0.03), and the mean value of the horizontal distance from the point “O” to the anatomic mark line was (3.43 ± 1.41) mm. The mean value of the vertical distance between point “O” and the upper edge of the sacrum was (6.10 ± 1.05) mm, as presented in Table 5.

**Table 5:** Positional relationship of the most secure operating center point relative to the center of the L5 pedicle and the upper edge of the sacrum at L5-S1

Side	ED/EF	DO (mm)	OP (mm)
Right	0.78 ± 0.03	3.31 ± 1.37	6.05 ± 1.07
Left	0.77 ± 0.03	3.55 ± 1.46	6.15 ± 1.03
Mean	0.77 ± 0.03	3.43 ± 1.41	6.10 ± 1.05

Values are expressed as mean ± SD

The point “O” represents the most secure operating center during the PETLIF procedure

The point “D” represents the intersection of the horizontal line of the point “O” with the vertical line passing through the center point of the L5 pedicle; values of the “ED/EF” are the ratio of the distance from the point “D” to the center point of the L5 pedicle to the vertical distance from the center point of the L5 pedicle to the upper edge of the sacrum; values of the “DO” are the horizontal distance from the point “O” to the vertical line passing through the center point of the L5 pedicle; values of the “OP” are the distance from the point “O” to the upper edge of the sacrum

## Discussion

The previous literature has reported a 2–3.5% chance of irritation or injury to the nerve roots with minimally invasive spinal surgery via the transforaminal approach [23–25]. Familiarity with the anatomy of the Kambin's triangle helps to avoid damage to nerve roots. Currently, most of the basic anatomic studies related to the Kambin's triangle are performed on cadavers, which not only have a small sample size but also have limitations that cannot be selected for the gender, age, and ethnicity of the specimens. Before measuring the relevant data on cadavers, it is necessary to preserve and process the specimen. Immersion of cadavers in formalin will cause deformation of the soft tissues, which in turn will change the size of the Kambin's triangle. In the process of exposure of the Kambin's triangle, the soft tissue around the intervertebral foramen and the nerve roots will be more or less damaged, which can easily lead to the displacement of the nerve roots and dural sac. There is a certain discrepancy between the anatomic data obtained from the cadaver and the actual anatomical size [26]. Thus, for such a narrow anatomic structure of the Kambin's triangle, the use of cadavers for basic anatomic research will produce errors of varying degrees. Compared with the cadaver-based research, the anatomic data of the Kambin's triangle based on imaging are more accurate, less distinct from the living body, and easier to achieve sufficient sample sizes. In the imaging examination, CT has an advantage in the ability to distinguish the bony structures, while MRI has a stronger ability to distinguish the soft tissue [27].

The imaging-based research about the Kambin's triangle anatomical data is not uncommon. But most of these focused on the detailed anatomy of the spine and did not consider the possibility of a foraminoplasty to resect a part of the SAP at the time of surgery. In the absence of the SAP resection, the limitation of the operating space by the boundaries of the Kambin's triangle will arise primarily from the SAP and the exiting nerve root, and the measurement of such studies often needs to be based on the CT scan, either on the sagittal projection of the three-dimensional anatomical corridor or on the two-dimensional image after rotation to a certain angle. Katsuhisa et al. measured based on 3DCT/MRI fusion imaging that the average distance between the exiting nerve roots and the SAP reached the maximum at L4–L5 when the image was rotated by 60° and was only (5.82 ± 2.20) mm [27]. The literature of Zhang et al. conducted research based on cadavers and CT, which pointed out that the average distance from the exiting nerve root to the SAP gradually increased from L1–L2 to L5–S1, with a maximum of (5.77 ± 0.56) mm [28]. Obviously, the size of the Kambin's triangle obtained by this method of measurement is small, and safe implantation of the cage with the 10 mm transverse diameter in such a narrow space is challenging. To avoid nerve root injury, foraminoplasty should be performed to dilate the operating space prior to cage implantation through the Kambin's triangle [20].

At present, foraminoplasty is routinely performed during the PETLIF. The circular saw is used to resect a portion of the SAP to obtain sufficient operating space under the endoscopy, and the size of the excision is dependent on the actual condition of the patient. Therefore, when acquiring basic anatomic data of the Kambin's triangle that corresponds to the PETLIF surgical technique, there is no need to consider the bone boundary, the SAP, and measurements in the sagittal plane are not necessary. The limitation of the operational space by the boundaries of projection of the Kambin's triangle on the coronal plane mainly comes from the exiting nerve root, the dural sac, and part of the traversing nerve root, which are also important anatomic structures that we should try to avoid injury during the surgical procedure. Hardenbrook et al., regardless of the SAP, measured the average value of the area of the Kambin's triangle to be 183 mm^2^, with a maximum of 219 mm^2^ at L5–S1. The authors considered the safe zone to be a trapezium bounded by the widths of the superior and inferior pedicles between the exiting and traversing nerve, this had an average area of 199mm^2^, and the average distance between the upper edge of the inferior pedicle and the exiting nerve roots was longer than 10 mm, to prove the feasibility of cage implantation through the Kambin's triangle [29]. On the other hand, the region between the exiting nerve root and the dural sac in the proximal part of the coronal projection of the Kambin's triangle is too narrow, which is difficult to utilize this region during the PETLIF, and the height of the intervertebral space also limits the size of the cage implanted. Thus, we believe that the trapezoid formed by transecting the coronal projection of the Kambin's triangle with the horizontal line of the inferior endplate of the superior vertebral body at the target intervertebral space is the actual secure region that can be used during the PETLIF, and we define this trapezoidal area as the secure region of the operation during the PETLIF procedures.

The surgical procedure for the PETLIF described in this study is as follows: After patient preparation, the endoscopic channel was punctured through the intervertebral foramina, and a circular saw was used to excise a portion of the SAP to expose the Kambin's triangle. Under the direct endoscopic-assisted vision, the intervertebral disk was excised, and bone grafting was performed after the endplate treatment. The expandable fusion cage filled with bone fragments was then implanted to restore the height of the intervertebral space. The vertebral body was finally fixed with the technique of the percutaneous posterior pedicle screw internal fixation. The technique of PETLIF integrates the technology of channel, endoscopy, expandable fusion cage, and percutaneous nailing, which has the advantages of reducing skeletal muscle damage, decreasing intraoperative blood loss, and shortening patient recovery time [30–33]. In the early days, the percutaneous endoscopic techniques for lumbar interbody fusion were used by Frederic et al. and did not perform the foraminoplasty during surgery, nor did they use the expandable fusion cage, reporting up to 36% of the complications rate related to surgery. Although they questioned this surgical technique, they also pointed out that adequate preoperative imaging assessment to localize the nerve roots and compare cage size and the operating space may help to avoid the occurrence of surgical complications [34]. In actual clinical work, the majority of patients requiring lumbar interbody fusion are accompanied by the diseases that change the size of the Kambin's triangle, such as degenerative lumbar disease, lumbar spondylolisthesis, and unstable spinal stenosis. Even the Kambin's triangle in healthy individuals may be relatively narrow because of anatomical variation, and the position may also vary the size of the Kambin's triangle [35, 36]. It is important to consider these important factors that may cause intraoperative nerve damage. Therefore, to avoid intraoperative nerve root injury, it is necessary to use the clearest and most accurate imaging examination to assess the patient's condition before surgery.

The MRN of the lumbar nerve roots is the best imaging technique for viewing nerve roots by fully distinguishing the anatomical structures such as the ligaments, intervertebral disks, and soft tissues while allowing for a clearer visualization of the dural sac and the nerve root, exhibiting accurately the course of the nerve roots and its relation to the adjacent anatomic structures [22]. This imaging examination allows the morphology of the Kambin's triangle to be visualized and helps to quantitatively assess the size of the secure region and individualize the feasibility of the surgical intervention. Therefore, it is of practical clinical significance to perform the MRN before surgery. To avoid nerve root injury and obtain accurate anatomical data of the Kambin's triangle corresponding to the current PETLIF surgical technique. In this study, the safety of cage implantation in each lumbar intervertebral space was discussed based on the measurement results of the MRN images, and a novel method of intraoperative localization was proposed.

The width of the cage implanted during the PETLIF is limited by the inner and outer boundaries of the secure region since these two boundaries are the nerve roots and the dural sac which focus on the prevention of injury during surgery. To assess safety, we use the measured width of the secure region as the primary observational indicator. Because the height of the cage is limited by the distance of the endplates of the upper and lower vertebral bodies of the target intervertebral space, we used the measured height of the secure region as a secondary observational indicator. At the same time, the area of the secure region reflects the size of the space that can be operated during the PETLIF and also serves as a secondary observational indicator.

The PETLIF was performed on all lumbar segments as described in Said's case series report, and 2 of the 60 patients complained of residual numbness postoperatively, and although the fusion segments of these two patients were not specifically described in the article, we speculate that it may be related to the narrower intraoperative manipulation space of the high lumbar intervertebral space [37]. Our study has shown that the L1–L2 has the smallest operating space among all lumbar segments, and all the observational indicators are smaller than the corresponding size of the cage. The primary observational indicator at the L2–L3 is less than the cage width, and the secondary observational indicators are greater than the corresponding size of the cage, but the difference is not great. Therefore, we believe that the maneuverable space during surgery is still small at L2–L3. At the same time, the safety evaluation of the primary observational indicators of these two segments of intervertebral space is considered high risk, so we believe that the operating space is narrow at the L1–L2 and L2–L3, and the nerve roots need to be stretched to a certain extent when the cage is implanted. When performing surgical procedures in both of these two intervertebral spaces, the surgeon must reinforce the fineness of the operation, handle it with care, and try to avoid damage to the nerve roots.

Single-segment fusion of L3-L4 using the PETLIF was recommended in the previous literature [14]. But in our results, the average width of the secure region at L3-L4 was (10.22 ± 1.63) mm, which was longer than the cage width, but this difference was smaller than 0.5 mm. The safety of the primary observational indicator was only 53.57%, which was assessed as medium risk. In contrast, the safety of the two secondary observational indicators reached 77.38% and 95.24%, respectively, which were assessed as low risk, and the average area and height were larger than the size of the cage. These results indicate that the risk of nerve root injury was not low when the PETLIF was performed in this segment, but that there was also a certain space for surgical operation. In addition, given that the anatomic variation in the nerve roots is also an important factor in the injury of the nerve roots during surgery, and the branching nerve often originates from the L4 nerve root [38]. Therefore, we believe that an adequate imaging examination should be performed preoperatively when the PETLIF is performed at L3–L4 to accurately localize the position and course of the nerve roots and to compare the size of the cage with the data of the secure region defined by us. Only in this way can the safety of the surgery be guaranteed and the risk of nerve root injury be reduced.

At L4–L5 and L5–S1, the mean value of each observational indicator was greater than the size of the cage, and the assessment of the safety of each indicator was low risk. The safety of the primary observational indicator at L4–L5 was as high as 96.43%. Therefore, our observations suggest that in both of these segments, particularly at the L4–L5, there is sufficient operating space for PETLIF, and the risk of nerve root injury is low. Surgeons should not be overly concerned about nerve root injury at L4–S1 during the surgery. Furthermore, we have also observed that the distance between the exiting nerve root and the traversing nerve root at the L5–S1 in the sagittal plane is significantly longer than that of the other lumbar segments, although it has not been measured, we believe that the actual surgical safe operation space at this segment in three-dimensional space may be larger than what we have measured if the interference of the bone on the surgical approach is not taken into account.

In conclusion, with the downward movement of the target intervertebral space segment at L1–L5, the safe operating space of the PETLIF gradually expands, and the surgical safety gradually increases; although the width of the secure region at L5-S1 is slightly decreased, the safety of the implanted cage is still guaranteed. In the case of high-level lumbar intervertebral spaces, the operating space can be expanded by performing pedicle screw fixation to stretch the intervertebral space and applying a nerve retractor to moderately stretch the nerve roots, and the size of the expandable fusion cage can be further reduced to improve the surgical safety and reduce the risk of nerve roots injury.

### A new method for locating the most secure operating point during the PETLIF

Because of the difficulty in accurately judging the position of nerve roots during the surgery and because of the narrowness of the Kambin's triangle, the PETLIF technique requests extremely high standards on the surgeon’s intraoperative positioning capability. In actual clinical work, the surgeon can only rely on the surgical experience and preoperative imaging data to localize the approximate position of the nerve roots, and locating the center of the Kambin's triangle is difficult, especially for those young inexperienced doctors, who need a lot of practice to master positioning skills. Based on the basic anatomical study of the Kambin's triangle, we first proposed the method of positioning the safest point of entry into the intervertebral space during the PETLIF to guide the positioning operation and improve surgical safety. This could help surgeons to master positioning skills more rapidly so that the surgical techniques of the PETLIF can be better promoted and developed.

With the downward movement of the target intervertebral space segment, the ratio of “AC/AB” was found to increase from (0.57 ± 0.04) to (0.63 ± 0.04) at L1–L5. That is to say, the horizontal line passing through the point “O” was increasingly vertical away from the center of the pedicle of the upper vertebral body and was closer to the center of the pedicle of the lower vertebral body at the target intervertebral space. But, the difference in the ratio of “AC/AB” between each segment was not large, and the mean value was around 0.6 at each target segment. The point “O” was located on the medial side of the anatomic mark line and the average horizontal distance from the anatomic mark line was gradually increased from L1–L2 to L4–L5, which were (0.48 ± 0.67) mm, (1.20 ± 0.89) mm, (2.72 ± 1.01) mm, and (3.69 ± 1.47) mm, respectively. Applying the above results to practice, the specific positioning process is as follows: The results obtained in the present study indicate that for each target segment at L1-L5, the center points of the upper and lower pedicles on the measured side are located with intraoperative fluoroscopy. A horizontal line is then drawn inward at the midpoint and downward about 3/5 of the line segment connecting the two points, and different distances are positioned inward on the horizontal line depending on the different segments, so as to obtain the accurate positioning of the most secure operating center point during PETLIF procedures.

At the L5-S1, due to the anatomic inclination of the sacrum, the center point of the pedicle of the S1 vertebral body cannot be clearly located, and the pedicle of the L5 on the measured side and the sacrum are considered to be the osseous anatomical landmarks. The mean value of the ratio of “ED/EF” was (0.77 ± 0.03). The point “O” was also located medial to the anatomic mark line and the average horizontal distance was (3.43 ± 1.41) mm. The average distance from the point “O” to the upper edge of the sacrum was (6.10 ± 1.05) mm. Applying the above results to practice, the specific positioning process is as follows: The center point of the L5 pedicle on the measured side is located with intraoperative fluoroscopy, and a vertical line is drawn across this point to intersect with the upper edge of the sacrum. A horizontal line is then drawn inward at the midpoint and downward about 4/5 of the line segment. Position approximately (3.43 ± 1.41) mm inward to obtain the most secure operating center point during PETLIF procedures.

Finally, the point “O” is used as the positioning point for the insertion of the sleeve, and the operative route is established by perforation to complete the surgical procedure. In this method of positioning, intraoperative fluoroscopy is used to obtain the osseous anatomical landmarks (the center of the pedicles and the upper edge of the sacrum), combined with preoperative MRN examination to accurately localize the point “O.” Then, regarding the “O” point as the positioning point to insert the sleeve and establish an operation channel to obtain a safe operating space with a sufficient distance from the nerve roots and the dural sac, to reduce the risk of nerve root damage during the SAP resection and the cage implantation and to make the surgical procedure safer.

In addition, in the process of retrospective observation, we found that the secure region was almost always a trapezoidal structure with a narrow top and a wide bottom on the coronal plane of the MRN. In the absence of anatomical variation or pathological changes, the positioning point of the insertion of the sleeve can be relatively downward, away from the dural sac and the nerve roots around the point “O.” However, there were very few secure regions with trapezoidal structures with a broad top and a narrow bottom, this again serves as a reminder that it is very important to perform an individualized assessment of patients with the MRN of the lumbar nerve roots preoperatively.

### Limitations

This study also has certain limitations. Firstly, this study is a single-center, retrospective, NRN-based study; therefore, further prospective multicenter studies with a large sample size are required to provide more convincing results. Second, the imaging data with obvious lumbar degenerative changes were excluded from the present study. In practice, however, those patients who need to receive the PETLIF are often accompanied by lumbar degenerative changes. The conclusion of this study has limitations for patients with obvious stenosis of the lumbar intervertebral space. Clinically, preoperative planning should be performed with real-time imaging data. In addition, the clearest level showing the course of the exiting nerve root was selected for measurement, but the exiting nerve root at L5–S1 was not at the same level as the traversing nerve root, and multiple levels of repeated measures were required. Despite our repeated confirmation of the position of the boundary of the Kambin's triangle, this may still lead to some errors. Lastly, due to the irregular morphology of the lower lumbar pedicles, particularly the pedicles of the L5 vertebral, on coronal imaging data, as well as the limited resolution of the bone by MRI imaging techniques, we have difficulty locating the central point of the pedicles, which may also cause certain errors. The effectiveness and practicability of the new method for locating the most secure operating center point can be further evaluated based on CT/MRI fusion images and a prospective randomized controlled study.

## Conclusions

The MRN allows clearer and more accurate visualization of the nerve roots, and the basic anatomic study of the Kambin's triangle based on this technology is of practical clinical significance. In the current study, it is believed that, during PETLIF, the cage implantation is the safest at L4–L5, followed by L5–S1; L1–L2 and L2–L3 are more likely to cause nerve root injury, and L3–L4 is not less likely. To improve safety, a comprehensive individualized imaging assessment should be performed before surgery. This study also provides an easy method of intraoperative localization, which helps avoid nerve root injury.

## Data Availability

The datasets used and analyzed during the current study are available from the corresponding author upon reasonable request.

## References

[1] Mummaneni PV, Dhall SS, Eck JC, Groff MW, Ghogawala Z, Watters WR, Dailey AT, Resnick DK, Choudhri TF, Sharan A, Wang JC, Kaiser MG (2014). Guideline update for the performance of fusion procedures for degenerative disease of the lumbar spine. J Neurosurg Spine.

[2] Mobbs RJ, Phan K, Malham G, Seex K, Rao PJ (2015). Lumbar interbody fusion: techniques, indications and comparison of interbody fusion options including PLIF, TLIF, MI-TLIF, OLIF/ATP. LLIF and ALIF J Spine Surg.

[3] Foley KT, Lefkowitz MA (2002). Advances in minimally invasive spine surgery. Clin Neurosurg.

[4] Lener S, Wipplinger C, Hernandez RN, Hussain I, Kirnaz S, Navarro-Ramirez R, Schmidt FA, Kim E, Hartl R (2020). Defining the MIS-TLIF: a systematic review of techniques and technologies used by surgeons worldwide. Global Spine J.

[5] Ge DH, Stekas ND, Varlotta CG, Fischer CR, Petrizzo A, Protopsaltis TS, Passias PG, Errico TJ, Buckland AJ (2019). Comparative analysis of two transforaminal lumbar interbody fusion techniques: open TLIF versus Wiltse MIS TLIF. Spine.

[6] Lee SG, Ahn Y (2021). Transforaminal endoscopic lumbar discectomy: basic concepts and technical keys to clinical success. Int J Spine Surg.

[7] Macki M, Hamilton T, Haddad YW, Chang V (2021). Expandable cage technology-transforaminal, anterior, and lateral lumbar interbody fusion. Oper Neurosurg.

[8] Patel DV, Yoo JS, Karmarkar SS, Lamoutte EH, Singh K (2019). Interbody options in lumbar fusion. J Spine Surg.

[9] Tumialan LM, Madhavan K, Godzik J, Wang MY (2019). The history of and controversy over Kambin's triangle: a historical analysis of the lumbar transforaminal corridor for endoscopic and surgical approaches. World Neurosurg.

[10] Fanous AA, Tumialan LM, Wang MY (2019). Kambin's triangle: definition and new classification schema. J Neurosurg Spine.

[11] Min JH, Kang SH, Lee JB, Cho TH, Suh JK, Rhyu IJ (2005). Morphometric analysis of the working zone for endoscopic lumbar discectomy. J Spinal Disord Tech.

[12] Ao S, Zheng W, Wu J, Tang Y, Zhang C, Zhou Y, Li C (2020). Comparison of Preliminary clinical outcomes between percutaneous endoscopic and minimally invasive transforaminal lumbar interbody fusion for lumbar degenerative diseases in a tertiary hospital: Is percutaneous endoscopic procedure superior to MIS-TLIF? A prospective cohort study. Int J Surg.

[13] Xue YD, Diao WB, Ma C, Li J (2021). Lumbar degenerative disease treated by percutaneous endoscopic transforaminal lumbar interbody fusion or minimally invasive surgery-transforaminal lumbar interbody fusion: a case-matched comparative study. J Orthop Surg Res.

[14] Wu J, Liu H, Ao S, Zheng W, Li C, Li H, Pan Y, Zhang C, Zhou Y (2018). Percutaneous endoscopic lumbar interbody fusion: technical note and preliminary clinical experience with 2-year follow-up. Biomed Res Int.

[15] Wang MY, Grossman J (2016). Endoscopic minimally invasive transforaminal interbody fusion without general anesthesia: initial clinical experience with 1-year follow-up. Neurosurg Focus.

[16] Kolcun J, Brusko GD, Basil GW, Epstein R, Wang MY (2019). Endoscopic transforaminal lumbar interbody fusion without general anesthesia: operative and clinical outcomes in 100 consecutive patients with a minimum 1-year follow-up. Neurosurg Focus.

[17] Hirayama J, Hashimoto M, Sakamoto T (2020). Clinical outcomes based on preoperative Kambin's triangular working zone measurements on 3D CT/MR fusion imaging to determine optimal approaches to transforaminal endoscopic lumbar diskectomy. J Neurol Surg A Cent Eur Neurosurg.

[18] Lertudomphonwanit T, Keorochana G, Kraiwattanapong C, Chanplakorn P, Leelapattana P, Wajanavisit W (2016). Anatomic considerations of intervertebral disc perspective in lumbar posterolateral approach via Kambin's triangle: cadaveric study. Asian Spine J.

[19] Khandge AV, Sharma SB, Kim JS (2021). The evolution of transforaminal endoscopic spine surgery. World Neurosurg.

[20] Ishihama Y, Morimoto M, Tezuka F, Yamashita K, Manabe H, Sugiura K, Takeuchi M, Takata Y, Sakai T, Maeda T, Nagamachi A, Sairyo K (2022). Full-endoscopic trans-Kambin triangle lumbar interbody fusion: surgical technique and nomenclature. J Neurol Surg A Cent Eur Neurosurg.

[21] Pairaiturkar PP, Sudame OS, Pophale CS (2019). Evaluation of dimensions of Kambin's triangle to calculate maximum permissible cannula diameter for percutaneous endoscopic lumbar discectomy : A 3-dimensional magnetic resonance imaging based study. J Korean Neurosurg Soc.

[22] Wang HL, Jiang JY, Lv FZ, Yang SD, Ma X, Chen WJ, Ma XS, Xia XL, Wang LX (2014). Magnetic resonance neurography in analysis of operative safety of transforaminal lumbar interbody fusion in Chinese subjects. Orthop Surg.

[23] Sairyo K, Matsuura T, Higashino K, Sakai T, Takata Y, Goda Y, Suzue N, Hamada D, Goto T, Nishisho T, Sato R, Tsutsui T, Tonogai I, Mineta K (2014). Surgery related complications in percutaneous endoscopic lumbar discectomy under local anesthesia. J Med Invest.

[24] Epstein NE (2016). More nerve root injuries occur with minimally invasive lumbar surgery: Let’s tell someone. Surg Neurol Int.

[25] Zhou C, Zhang G, Panchal RR, Ren X, Xiang H, Xuexiao M, Chen X, Tongtong G, Hong W, Dixson AD (2018). unique complications of percutaneous endoscopic lumbar discectomy and percutaneous endoscopic interlaminar discectomy. Pain Physician.

[26] Arslan M, Comert A, Acar HI, Ozdemir M, Elhan A, Tekdemir I, Tubbs RS, Ugur HC (2012). Nerve root to lumbar disc relationships at the intervertebral foramen from a surgical viewpoint: an anatomical study. Clin Anat.

[27] Yamada K, Nagahama K, Abe Y, Hyugaji Y, Takahata M, Iwasaki N (2021). Morphological analysis of Kambin's triangle using 3D CT/MRI fusion imaging of lumbar nerve root created automatically with artificial intelligence. Eur Spine J.

[28] Zhang L, Yang J, Hai Y, Yin P, Ding Y, Xu C, Gao H (2020). Relationship of the exiting nerve root and superior articular process in Kambin's triangle: assessment of lumbar anatomy using cadavers and computed tomography imaging. World Neurosurg.

[29] Hardenbrook M, Lombardo S, Wilson MC, Telfeian AE (2016). The anatomic rationale for transforaminal endoscopic interbody fusion: a cadaveric analysis. Neurosurg Focus.

[30] Yin P, Gao H, Zhou L, Pang D, Hai Y, Yang J (2021). Enhanced recovery after an innovative percutaneous endoscopic transforaminal lumbar interbody fusion for the treatment of lumbar spinal stenosis: a prospective observational study. Pain Res Manag.

[31] Nagahama K, Ito M, Abe Y, Murota E, Hiratsuka S, Takahata M (2019). Early clinical results of percutaneous endoscopic transforaminal lumbar interbody fusion: a new modified technique for treating degenerative lumbar spondylolisthesis. Spine Surg Relat Res.

[32] Jin M, Zhang J, Shao H, Liu J, Huang Y (2020). Percutaneous transforaminal endoscopic lumbar interbody fusion for degenerative lumbar diseases: a consecutive case series with mean 2-year follow-up. Pain Physician.

[33] Nakamura S, Ito F, Ito Z, Shibayama M (2020). Methods and early clinical results of percutaneous lumbar interbody. Fusion Neurospine.

[34] Jacquot F, Gastambide D (2013). Percutaneous endoscopic transforaminal lumbar interbody fusion: Is it worth it?. Int Orthop.

[35] Ozer AF, Suzer T, Can H, Falsafi M, Aydin M, Sasani M, Oktenoglu T (2017). Anatomic assessment of variations in Kambin's triangle: a surgical and cadaver study. World Neurosurg.

[36] Botanlioglu H, Aydingoz O, Kantarci F, Kaynak G, Guven MF, Ertan S (2015). Positional alterations of the Kambin's triangle and foraminal areas in the lumbosacral region. Acta Orthop Traumatol Turc.

[37] Osman SG (2012). Endoscopic transforaminal decompression, interbody fusion, and percutaneous pedicle screw implantation of the lumbar spine: a case series report. Int J Spine Surg.

[38] Dindial R, Iwanaga J, Dumont AS, Tubbs RS (2021). Rare variation of the furcal nerve. Morphologie.

